# Written Communication From Pharmacy Benefit Managers: Is It Helpful?

**DOI:** 10.7759/cureus.50342

**Published:** 2023-12-11

**Authors:** Lily G Cessna, Lynne J Goebel

**Affiliations:** 1 Internal Medicine, Marshall University Joan C. Edwards School of Medicine, Huntington, USA

**Keywords:** beers list, drug-drug interactions, burnout, communications, pharmacy benefit managers

## Abstract

Introduction: Physicians are experiencing greater burnout due to excessive time spent on paperwork. Pharmacy benefit managers (PBMs) are adding to this problem by sending excessive mail to physicians. This study examined these mailed communications to determine their frequency and if the physician acted upon them. We hypothesized that few of these mailings would be helpful to the physician.

Methods:From July 2021 to May 2023, we collected all written communications from PBMs to a single geriatrics outpatient physician. We sorted this information by specific PBM, by communication category, if it resulted in an intervention, and if communications were repeated.

Results:We found that out of 263 communications, 17 (6%) resulted in interventions made by the physician. Notices of nonformulary prescriptions (35%, N=6/17) and drug-drug interactions (35%, N=6/17) (p=0.001) resulted in interventions most frequently. The Beers list notifications did not result in intervention. Forty-one percent (108/263) of communications were repeated, and almost half of these were for recommendations (N=52/108, 48%), a category that did not result in frequent interventions.

Conclusion:Our hypothesis that only a small number of communications were helpful to physicians was supported. A physician will more likely make interventions if the suggestion is regarding a nonformulary prescription or a drug-drug interaction. Interestingly, notification of a medication on the Beers list did not result in a geriatrician's action. Feedback to PBMs on which communications are most helpful may decrease the amount of paperwork received by physicians and aid in combating burnout.

## Introduction

Pharmacy benefit managers (PBMs) began in the late 1960s to assist private insurance companies in separating medical insurance coverage from prescription drug coverage [1]. Initially, they had administrative duties such as processing pharmacy claims and mail-service pharmacies, and they quickly evolved over the succeeding years into a more complex role [1]. PBMs are now responsible for formulary prescription management, where a PBM makes a list of drugs approved for reimbursement for their clients [2]. They perform utilization reviews detecting adverse reactions caused by multiple drugs prescribed to a patient, drugs that interfere with the patients' diseases, and over- and underuse of prescribed medications [2]. In addition, they endeavor to improve the quality of care for patients by employing medication therapy management where they work with patients to improve medication adherence and enroll chronically ill patients in disease management programs [2].

When a PBM changes a drug formulary, notices a problem during the utilization review, or detects a patient not adherent to their medication, they send this information to the patient's physician. This generates an abundance of paper mail for physicians. This paperwork increases when PBMs repeat themselves and send additional mail for the same issues detected. When physicians are already spending more time doing paperwork and less time with patients, these communications can take additional time away from patient care and contribute to burnout causing physicians to retire early. This becomes more concerning when the Association of American Medical Colleges estimates a shortage of up to 55,200 primary care physicians in the next 10 years [3]. This study aims to determine the frequency of communications from PBMs and which types of information from PBMs are most helpful to physicians. We hypothesize that only a small amount of the information is helpful to physicians.

## Materials and methods

This study was approved by the Marshall University Internal Review Board (approval number: 1789222). From July 2021 to May 2023, we collected all written communications from PBMs mailed to a single outpatient geriatrician. We included all communications about medications for patients seen at Hanshaw Geriatric Center located in Huntington, West Virginia, by this physician. Communications that were not medication-related were excluded from the review such as home visits for peripheral vascular disease testing, case management or home well-being assessment, and hospitalization notices.

Investigators entered the data into an Excel spreadsheet (Microsoft Corporation, Redmond, Washington, United States). We recorded the age, gender, date of communication, communication category, and name of the PBM. Communication categories included nonformulary notifications, drug-drug interactions, drug-disease interactions, recommendations, and the Beers list. Nonformulary prescriptions are prescriptions that are not included in the insurance company's covered medications. Drug-drug interactions are when two or more of the patient's medications are interacting. Drug-disease interactions are when a medication is interfering with a diagnosed disease. Recommendations are when the PBM suggests a medication for a current disease (e.g., a statin for a diabetic patient). The Beers list is a list of medications that are potentially inappropriate for use in older adults. The physician wrote on a communication if it resulted in an intervention, and this was noted in the spreadsheet. We also made note of those communications that were repeated for the same patient for the same reason.

We analyzed the communications using basic descriptive statistics (Stata Statistical Software: Release 17 (2021; StataCorp LLC, College Station, Texas, United States)). A comparison of intervention and non-intervention groups used Fisher's exact test when appropriate. An alpha level of less than 0.05 determined statistical significance.

## Results

We analyzed 263 communications among 83 patients from a physician's panel of 431 patients. The median age for all participants in the study was 79 years. The group that received interventions from the physician and the group that did not receive interventions from the physician were comparable in median age at 77 and 80 years, respectively (p=0.89). The percentage of males in this study was 11% (N=28/263). Gender did not differ significantly between groups with 6% (N=1/17) males in the intervention group and 11% (N=27/246) males in the non-intervention group (p=1.00).

**Figure 1:**
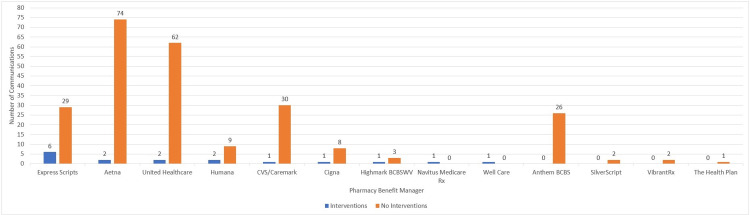
Number of written communications by PBMs and number of interventions PBMs: pharmacy benefit managers

In the 22-month period, the physician received 263 written communications. Aetna (Hartford, Connecticut, United States) sent the most communications (N=76, 29%), followed by United Healthcare (Minnetoka, Minnesota, United States) (N=64, 24%) and Express Scripts (St. Louis, Missouri, United States) (N=35, 13%) (Figure 1).

Communications from Express Scripts resulted in the most interventions made (N=6/35, 17%). Express Scripts communications most frequently acted upon included drug-drug interactions (N=3/6, 50%), drug-disease interactions (N=2/6, 66%), and recommendations (N=1/6, 17%). This was trending towards significance (p=0.063). None of the 26 communications from Anthem Blue Cross Blue Shield (BCBS) (Indianapolis, Indiana, United States), SilverScript (Nashville, Tennessee, United States), and VibrantRx (San Diego, California, United States) resulted in an intervention by the physician. Only two of 76 (3%) communications from Aetna resulted in an intervention, and both were nonformulary notifications (p=0.002). Their most common communication requested drug recommendations based on disease present, and none of these resulted in an intervention. 

**Figure 2:**
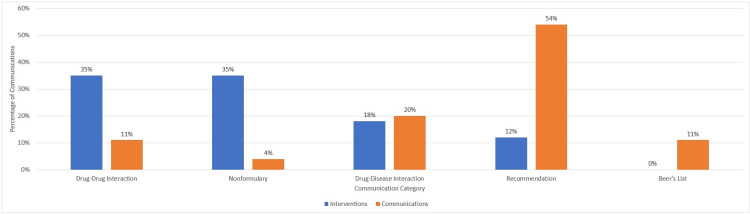
Percentage of written communications by communication category with and without interventions

The top two categories of communications overall were recommendations (e.g., a diabetic patient should be on a statin) (N=142, 54%) and drug-disease interactions (N=53, 20%) (Figure 2). 

Only 17/263 (6%) communications from PBMs resulted in an intervention from the physician. Communications about nonformulary (N=6/17, 35%) and drug-drug interactions (N=6/17, 35%) resulted in the most interventions, while the Beers list notifications resulted in no interventions (p=0.001).

**Figure 3:**
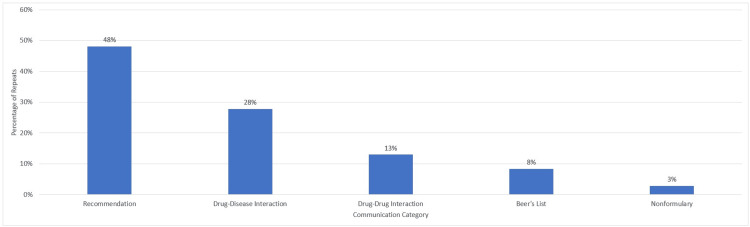
Percentage of repeats by communication category

There were 108 repeat communications. Recommendations had the most written communication repeats (N=52, 48%), while nonformulary had the least repeats (N=3, 3%) (Figure 3).

## Discussion

Our research supported our hypothesis that few communications between PBMs and primary care physicians led to interventions by the physician. A 2018 American Society of Clinical Oncology (ASCO) practice survey found that 10% of medical practices reported benefits when working with PBMs, while 75% reported PBMs interfered with patient care and getting work done [4]. Low perceived benefits and high patient care interruptions may cause physicians to neglect communications and lead to a low number of interventions.

Some communication categories such as nonformulary prescriptions and drug-drug interactions resulted in more interventions than other categories. One possible reason for the high intervention rate for nonformulary prescriptions could be the physician's perceived benefit for patient care. The average copay for formulary brand-name drugs is $10, while for nonformulary brand-name drugs, the cost is often much higher [5]. If a prescription is nonformulary, the insurance company will sometimes refuse to pay, leaving the cost burden on the patient. This can lead to nonadherence for the patient who is not able to afford the medication and frustration for the physician who is not able to help the patient. One way that the PBM may increase medication adherence and limit the paperwork burden on the physician is to communicate with the patient directly instead of mailing information to the physician [6]. Hodgkin et al. found that nonformulary prescriptions for antidepressants resulted in an 11% decrease in adherence to the prescriptions [7]. If, however, a physician changes the medication to one that the insurance company covers, this can allow for better adherence. Therefore, this type of communication can be seen as worthwhile. Sometimes, the PBM suggests alternate medications that are on their formulary. If they do not, then the physician must look up the formulary, something that takes physician time unless the physician has an employee who can help with this task.

The fact that drug-drug interactions resulted in the most interventions was not surprising. One explanation for this may be that increased or decreased levels of medication could lead to adverse side effects or decreased efficacy. Solberg et al. estimated from two large health plans that the frequency of drug-drug interactions was 6.2% per year [8]. Another study found that the most common of these interactions were serotonin syndrome, seizures, bleeding, and prolonged QT interval [9]. One of these commonly reported drug-drug interactions, QT interval prolongation, could lead to serious cardiac arrhythmia or death. This may be perceived as important enough to warrant looking for alternative drugs that do not have this interaction. Once again, if the PBM suggested alternate formulary medications that do not cause the interaction, physicians may welcome this information.

Older adults take a lot of medications. In fact, one study found that 53% of adults over the age of 65 took more than four medications in 2014 [10]. Another study in 2001 found that while 13% of the nation's population were geriatric, they accounted for over 33% of drug spending [11]. Deprescribing is a popular practice among geriatricians, and the Beers list of potentially inappropriate medications (PIMs) is one tool for identifying medications to be deprescribed [12]. Surprisingly, notification of medication on the Beers list led to no interventions in our study of a geriatrician's practice. Some of the reasons why no interventions were made in this category may be that patients have been taking these medications for years, are not suffering the potential side effects, or do not want to switch [12]. Primary care physicians have limited time with patients, and competing reasons for the visit do not always allow time to discuss changing these medications [13]. Primary care physicians also may not feel comfortable deprescribing medication originally prescribed by other subspecialty physicians who comanage their patients [13]. After publication of the updated Beers criteria in 2003, prescription of PIMs decreased from 22.5% to 15.1% showing that the Beers list is helpful [12]. Vandergrift et al. found that internists are more likely to prescribe PIMs compared to geriatricians [14]. So, internists who are not as familiar with the Beers list may benefit from these PBM communications more than a geriatrician.

Although Aetna sent the most communications, their information resulted in few interventions. They sent the most communications about recommendations that were based on the presence of a disease and the guideline to add a medication, such as adding statins in patients with diabetes, but no interventions were made. Many times, physicians have already addressed this with the patient, and either the patient tried the medication and had side effects or refused to take the medication. This is not known by the PBM and may result in repeated communications for the same problem. Some PBMs have a sheet that the physician can fill out that would notify the PBM about this. However, it requires physician time to review the chart, look up the reason, and fill out the form, with limited benefit to the patient and unknown effect on limiting repeat communications. Express Scripts sent the highest percentage of communications that led to interventions, most of which were drug-drug or drug-disease interactions.

According to one study, physicians spend 27% of their time in clinic with patients and 49% on desk work [15]. Spending more time on administrative duties and less time with patients leads to increased physician burnout and more physicians considering early retirement. A study from 2011 to 2014 found that 50% of physicians reported one symptom of burnout with one of the leading causes of burnout being paperwork [16]. Importantly, a systematic review of reasons physicians retire early found that burnout and excessive workload were cited most frequently [17]. Knowing which communications are most helpful to physicians may help PBMs streamline the number of communications and lead to better use of their resources while limiting communication alert fatigue. This may help decrease physician workload and reduce burnout and considerations for early retirement.

A limitation of this study is it only includes data from only one physician. Including other physicians would increase the number of communications and possible interventions in the study. In addition, including physicians in other specialties and geographical regions may show different results as they may not receive the same number and type of PBM communications.

## Conclusions

This small study of PBM communications to a geriatrician showed that few communications were helpful. PBMs should focus on nonformulary prescriptions and drug-drug interactions since these categories are most likely to result in physician action. Categories such as suggestions for adding medication based on a known diagnosis were not seen as helpful. PBMs often do not have enough information about the situation to know if these interventions are appropriate for the individual patient, and when their suggestions are not followed, they tend to send repeat notifications. Excessive paperwork can contribute to burnout and may lead some physicians to retire early contributing to the physician shortage problem. In fact, many physicians may suffer from repetitive alert fatigue, destroying all notifications and missing the few that are potentially helpful. 
